# Prevalence of anxiety and depression among Lebanese women using oral contraceptives: a cross-sectional study

**DOI:** 10.1186/s12905-024-02897-4

**Published:** 2024-01-18

**Authors:** Kamel Jaafar, Elias Nabhan, Rama Daoud, Zeina Nasser

**Affiliations:** 1 Faculty of Medical Sciences, Department of Obstetrics and Gynecology, Lebanese University, Hadath, Lebanon; 2 Neurosciences Research Center, Lebanese University, Hadath, Lebanon; 3 Faculty of Medical Sciences, Department of Internal Medicine, Cardiology Division, University of Balamand, Beirut, Lebanon; 4 Faculty of Medical Sciences, Lebanese University, Hadath, Lebanon; 5 Faculty of Medical Sciences, Neurosciences Research Center, Lebanese University, Hadath, Lebanon

**Keywords:** Anxiety, Depression, Oral contraception, Women, Lebanon

## Abstract

**Background:**

Oral contraceptives (OCs) are used worldwide, including Lebanese women. However, the association between OCs use and anxiety or depression remains unclear. This study aims to assess the prevalence of anxiety and depression among Lebanese women using oral contraceptive pills and investigate the differential impact of combined oral contraceptives (COCs) versus progestogen-only pills (POPs) on mental health outcomes.

**Methods:**

A cross-sectional study was conducted among a sample of Lebanese women using OCs between January and March 2023. Nine hundred nighty seven out of the 2051 women who took part in the survey met our criteria and were included in this study. Data on anxiety and depression were collected using validated and reliable scales, the Arabic versions of the Generalized Anxiety Disorder-7 Questionnaire (GAD-7) and the Patient Health Questionnaire-9 (PHQ-9). Statistical analyses, including multivariate analysis, were performed to assess the association between OCs type (COC vs. POP) and anxiety/depression.

**Results:**

The prevalence of anxiety and depression among Lebanese women taking OCs was found to be 39.9% and 64.3%, respectively. Furthermore, the study revealed that POP users had 2.8 times higher odds of developing anxiety (adjusted odds ratio OR_adj_ = 2.8 with 95% confidence interval CI of 1.770 to 4.435) *p*-value < 0.001 and 9.2 times higher odds of developing depression (adjusted odds ratio OR_adj_ = 9.2 with 95% confidence interval CI of 5.790 to 14.506) *p*-value < 0.001 compared to COC users.

**Conclusion:**

The results of this study shed light on the elevated prevalence of anxiety and depression among Lebanese women using OCs and emphasized the varying effects of COCs and POPs on their mental health outcomes. Further research is needed to comprehensively understand this association, considering both the dosage and specific type of oral contraceptive to improve the overall well-being of women using these contraceptives.

## Background

Oral contraceptives are widely used worldwide, with over 100 million female users [1]. Their popularity can be attributed to factors such as accessibility, cost-effectiveness, and ease of use [2]. In Lebanon, OCs are the preferred method of contraception for 22.2% of women, as reported in a nationwide survey conducted in 2009 [3]. However, the choice of contraception depends on various factors, including age, general health, sexual activity, number of partners, fertility intentions, and medical history [4].

OCs are hormonal formulations that contain a combination of estrogen and progestin or progestin alone. They work by suppressing the production of luteinizing hormone (LH) and follicle-stimulating hormone (FSH), thereby preventing conception [5]. When used correctly, OCs are highly effective, with a 99% success rate. However, they have been associated with certain adverse effects, including gallstones, hypertension, and an increased risk of thromboembolism, especially in smokers [6]. Depression was identified as a significant adverse consequence since the early 1960s, leading to a decline in OC usage [7, 8]. Nevertheless, the relationship and the certainty of this association remains debatable until now; with numerous studies in the literature investigating this correlation yet the findings have been varied [9–11].

Depression is a prevalent and debilitating condition affecting a significant portion of the Lebanese population [12]. Studies have reported depression rates of 59.7% in Lebanon, which are higher compared to other countries [13]. A similar study conducted in Saudi Arabia reported a depression prevalence of 30.6% among women using hormonal contraception and showed that certain types of contraceptives have been associated with a higher risk of mood disturbances and depression compared to other birth control methods [14]. Overall, women using oral contraceptive pills are more likely to experience depressive symptoms than non-users [15].

A prospective cohort study involving over one million women found that hormonal contraception use increased the risk of subsequent antidepressant use by 23% [16]. Other research suggests that sex steroid hormones influence brain regions associated with emotion and cognition, and progesterone administration during hormone treatment can have negative mood effects [17, 18]. The metabolites of progesterone may affect the inhibitory system in the central nervous system, potentially contributing to mood disturbances [19]. Exogenous progestins have been found to increase monoamine oxidase levels, leading to lower serotonin levels and possible depression and irritability [20]. Fluctuations in estrogen levels have also been associated with depressive episodes, with women at risk for depression experiencing mood changes in response to these hormonal fluctuations [21].

While there have been numerous studies investigating the relationship between OC use and mood disorders, the findings have been varied. To the best of our knowledge, no studies have been conducted in Lebanon to assess the association between hormonal contraception and anxiety or depression. Therefore, this study aims to determine the prevalence of anxiety and depression among Lebanese women using oral contraceptive pills and investigate the differential impact of COCs versus POPs on mental health outcomes. By addressing this research gap, we aim to contribute to the understanding of the potential effects of hormonal contraceptives on anxiety and depression in the Lebanese population.

## Methods

### Study design and population

A community-based cross-sectional study, using an online survey, was conducted between January and March 2023. Our inclusion criteria consisted of women who were living in Lebanon, aged 18 to 45, using either progestin-only pills or combined oral contraceptives containing both estrogen and progesterone, and had no history of anxiety, depression, or any mental health condition.

We excluded participants who do not use hormonal contraceptive pills or employ other forms of hormonal contraceptives (such as intramuscular injections, vaginal rings, transdermal patches), not Lebanese, having multiple comorbidities (for example, diabetes mellitus, chronic renal disease, cardiac diseases…), currently taking medication that affects central nervous system function, with any history of seizures, menopausal women, those who had been diagnosed with cancer within the past 3 years and/or had an active neoplastic disease. Out of the 2051 women who took part in the survey, 997 met our criteria and were included in this study.

### Sample size calculation and data collection

The sample size was calculated with a confidence level of 95% which means z-score = 1.96, and a 5% margin of error, with a 29% expected prevalence of depression in the population based on previous study in KSA [14]. The estimated sample size was 317 participants.

An online questionnaire using Google Forms was distributed through social media using a snowball technique, in both Arabic and English versions. It consisted of several sections. The socio-demographic section covered various aspects, including age, gender, marital status, place of residency, monthly income, educational level, smoking habits, and alcohol consumption.

The obstetric and gynecological history section captured information such as the number of children, history of abortion, time since last delivery, current contraception use, reasons for using contraception, duration of use, and the specific type of contraception employed.

The mental health section incorporated two validated scales to assess the presence of anxiety and depression among the Lebanese population. Specifically, the Arabic versions of the Generalized Anxiety Disorder-7 Questionnaire (GAD-7) and the Patient Health Questionnaire-9 (PHQ-9) were included. These scales have demonstrated good psychometric properties when used with Arabic-speaking Lebanese psychiatric patients [22]. Permission was obtained from the author to utilize the Arabic versions of these scales in our study.

#### The Generalized Anxiety Disorder-7 (GAD-7)

The Gad-7 is widely used as a self-reporting scale to assess the symptoms of anxiety. It consists of 7 items that measure anxiety over the past 2 weeks. Items are rated on a 4-point Likert-type scale (0 = not at all, 1 = several days, 2 = more than half the days, 3 = nearly every day). The GAD-7 score is calculated by summing up the seven items with higher scores indicating a greater risk of anxiety. GAD-7 total score for the seven items ranges from 0 to 21. An individual with a score of 10 or above is diagnosed with anxiety [22]. The reliability of the scale among the current sample was excellent (Cronbach’s Alpha, α = 0.844).

#### The Patient Health Questionnaire-9 (PHQ-9)

The PHQ-9 is a brief self-report measure of 9 items, employed to assess and grade depression severity over the past 2 weeks. Responses ranged from 0 to 3 (0 = not at all, 1 = several days, 2 = more than half the days, 3 = nearly every day). Total scores, obtained by summing the responses to each item, range from 0 to 27. Cut-off score adopted in the present study is a score of 10 or above for an individual to be diagnosed with depression [22]. The reliability of the scale among the current sample was excellent (Cronbach’s Alpha, α = 0.885).

### Pilot study

The survey was pilot tested in a sample of 35 women, in person, to check the clarity and readability of all items. Participants did not report any problems in understanding the questionnaire. On average, the survey was completed within approximately 10 min. The data from the pilot study was removed from the final analysis.

### Ethical considerations

Ethical approval was obtained by the scientific research committee of the Neuroscience Research Center, Faculty of Medical Sciences at the Lebanese University, Participants will answer a yes–no question to confirm their willingness to participate voluntarily. All the necessary measures to safeguard participants’ anonymity and confidentiality of information were respected. Written informed consent was obtained from all the participants.

### Statistical analysis

Data coding, data cleaning, and analysis have been carried out by using IBM SPSS (Version 22.0, IBM SPSS, IBM Corp, USA). Cronbach’s alpha coefficients were calculated to indicate scale reliability. All categorical variables were reported as frequencies and percentages. Both bivariate and multivariable logistic regression analyses were performed to identify associated factors of depression and anxiety. The variables in bivariate analysis with *p*-value < 0.2 were entered into multivariable logistic regression. Adjusted odds ratio and their 95% confidence intervals were reported. The final logistic regression model was reached after ensuring the adequacy of our data using the Hosmer and Lemeshow test. The statistical significance level was set at *p*-value < 0.05 (two-sided).

## Results

### Characteristics of the study population:

Characteristics of the Study Population are shown in Table 1. Most of our participants were aged between 21 and 25 years (23.1%), 39.7% living in Beirut and 26.6% in Mount Lebanon. 61.1% of participants were married, 86.9% of them were in the middle economic class, 73.9% had higher education, 96.4% of them were non-alcoholics and 67.1% were non-smokers.

**Table 1:** Characteristics of the study population (*n* = 997)

Characteristic	Category	n (%)
**Age**	18–20	84 (8.4%)
	21–25	231 (23.1%)
	26–30	181 (18.1%)
	31–35	204 (20.4%)
	36–40	195 (19.5%)
	41–45	102 (10.2%)
**Place of residency**	Beirut	396 (39.7%)
	Mount Lebanon	265 (26.6%)
	South	89 (8.9%)
	North	121 (12.1%)
	Beqaa	126 (12.6%)
**Marital Status**	Single	363 (36.4%)
	Married	609 (61.1%)
	Divorced	25 (2.5%)
**Income per month**	Low	120 (12.0%)
	Middle	866 (86.9%)
	High	11 (1.1%)
**Educational Level**	Above high school	737 (73.9%)
	High School and below	260 (26.1%)
**Smoking**	No	669 (67.1%)
	Yes	328 (32.9%)
**Alcohol Consumption**	No	961 (96.4%)
	Yes	36 (3.6%)
**Number of children**	None	395 (39.6%)
	One Child	105 (10.5%)
	Two Children and more	497 (49.9%)
**Last Delivery**	None	401 (40.2%)
	Less than 3 months	59 (5.9%)
	More than 3 months	537 (53.9%)
**Abortion**	Never	764 (76.6%)
	Once	49 (4.9%)
	Twice or more	184 (18.5%)
**Type of oral Contraceptive**	POP	630 (63.2%)
	COC	367 (36.8%)
**Reason for OCP intake**	Contraception	497 (49.8%)
	Menstrual disorders	415 (41.6%)
	PCOS	85 (8.5%)
**Duration of OCP intake**	Less than or equal to 6 months	596 (59.8%)
	More than 6 months	401 (40.2%)

*n* frequency, *%* percentage, *OCP* oral contraceptive pill, *COC* combined oral contraceptive, *POP* progesterone only pill, *PCOS* polycystic ovarian syndrome

Regarding the obstetric part, 39.6% of participants had no children. Of those who had children, 49.9% had two or more children, 53.9% gave birth more than 3 months ago, and 76.6% had no history of abortion.

Among the sample, 63.2% (*n* = 630) reported POPs use, while 36.8% (*n* = 367) reported using COC. Approximately half of our participants 49.8%, used oral contraceptive pills for contraception, whereas 41.6%, and 8.5% used the pills for abnormalities in menstrual function and Polycystic ovary syndrome (PCOS) respectively. Furthermore, it was found that 59.8% (*n* = 596) of the participants had been using contraception for 6 months or less.

### The prevalence of anxiety and its association with the type of oral contraceptive

**Fig. 1:**
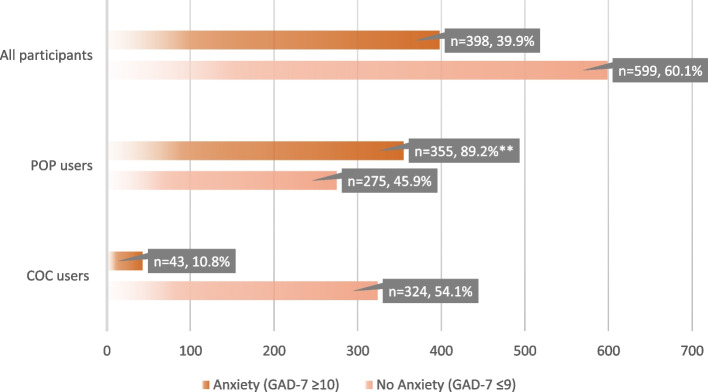
Anxiety prevalence among All participants, POP users, and COC users. ** Significant at *p*-value < 0.001. COC: combined oral contraceptive, POP: progesterone-only pills. GAD-7: Generalized Anxiety Disorder 7

Overall, four out of ten of the participants had anxiety 39.9% based on GAD-7 cut-off scores (≥ 10) (Fig. 1), and it was higher among participants taking POP 89.2% as compared to COC users 10.8% (Fig. 1).

### The association between anxiety and different population characteristics

The bivariate analysis revealed significant associations between various factors and anxiety among the participants. Age, current residency, marital status, income per month, smoking, reason for OCP intake, duration of OCP intake, type of oral contraceptive and depression were found to be associated with anxiety, *p*-value < 0.05. Anxiety didn’t differ statistically with education level, alcohol consumption, number of children, last delivery, and abortion, *p*-value > 0.05 (Table 2).

**Table 2:** Association between anxiety and population characteristics

	**Anxiety**		***P*-value**
	**No Anxiety n (%)**	**Anxiety n (%)**	
**Age**			
**18–20**	62 (10.4%)	22 (5.5%)	< 0.001^*^
**21–25**	159 (26.5%)	72 (18.1%)	
**26–30**	96 (16.0%)	85 (21.4%)	
**31–35**	106 (17.7%)	98 (24.6%)	
**36–40**	118 (19.7%)	77 (19.3%)	
**41–45**	58 (9.7%)	44 (11.1%)	
**Place of residency**			
**Beirut**	221 (36.9%)	175 (44.0%)	< 0.001^*^
**Mount Lebanon**	151 (25.2%)	114 (28.6%)	
**South**	44 (7.3%)	45 (11.3%)	
**North**	90 (15.0%)	31 (7.8%)	
**Beqaa**	93 (15.5%)	33 (8.3%)	
**Marital Status**			
**Single**	260 (43.4%)	103 (25.9%)	< 0.001^*^
**Married**	326 (54.4%)	283 (71.1%)	
**Divorced**	13 (2.2%)	12 (3.0%)	
**Income per month**			
**Low**	88 (14.7%)	32 (8.0%)	0.007^*^
**Middle**	505 (84.3%)	361 (90.7%)	
**High**	6 (1.0%)	5 (1.3%)	
**Educational level**			
**High school and below**	169 (28.2%)	91 (22.9%)	0.060
**Above high school**	430 (71.8%)	307 (77.1%)	
**Smoking**			
**No**	383 (63.9%)	286 (71.9%)	0.009^*^
**Yes**	216 (36.1%)	112 (28.1%)	
**Alcohol Consumption**			
**No**	575 (96.0%)	386 (97.0%)	0.411
**Yes**	24 (4.0%)	12 (3.0%)	
**Number of children**			
**None**	271 (4.4%)	124 (7.1%)	0.321
**1 child**	58 (16.9%)	47 (15.9%)	
**2 or more children**	270 (78.7%)	227 (76.9%)	
**Last Delivery**			
**None**	277 (4.7%)	124 (7.1%)	0.074
**Less than 3 months**	25 (7.4%)	34 (11.5%)	
**More than 3 months**	297 (87.9%)	240 (81.4%)	
**Abortion**			
**Never**	475 (63.4%)	289 (63.1%)	0.796
**Once**	24 (7.1%)	25 (8.5%)	
**Twice or more**	100 (29.5%)	84 (28.5%)	
**Reason for OCP intake**			
**Contraception**	212 (35.4%)	285 (71.6%)	< 0.001^*^
**Menstrual disorders**	328 (54.8%)	87 (21.9%)	
**PCOS**	59 (9.8%)	26 (6.5%)	
**Duration of OCP intake**			
**Less than or equal to 6 months**	390 (65.1%)	206 (51.8%)	< 0.001^*^
**More than 6 months**	209 (34.9%)	192 (48.2%)	
**Type of Oral Contraceptive**			
**POP**	275 (45.9%)	355 (89.2%)	< 0.001^*^
**COC**	324 (54.1%)	43 (10.8%)	
**Depression**			
**No**	327 (54.6%)	29 (7.3%)	< 0.001^*^
**Yes**	272 (45.4%)	369 (92.7%)	

*n* frequency, *%* percentage, *OCP* oral contraceptive pill, *COC* combined oral contraceptive, *POP* progesterone only pill, *PCOS* polycystic ovarian syndrome

^*^Significant at *p*-value < 0.05, *p*-values obtained using Pearson’s chi-square test

Table 3 showed the multivariate logistic regression. Our results showed that individuals living in Beqaa and North Lebanon regions had adjusted odds ratios of 0.248 (95% CI: 0.136–0.452) *p*-value < 0.001 and 0.304 (95% CI: 0.167–0.551) *p*-value < 0.001, respectively, therefore, were less likely to be anxious compared to those living in Beirut. Additionally, smokers were found to be less anxious compared to non-smokers, with an adjusted odds ratio of 0.362 (95% CI: 0.236–0.558) *p*-value < 0.001.

**Table 3:** Odds ratios with their 95% confidence intervals from the logistic regression analysis for anxiety

Anxiety						
	**Adjusted Odds Ratio (OR**_**adj**_**)**	**95% CI**	***P*-value**	**crude Odds Ratio (OR)**	**95% CI**	***P*-value**
**Age**						
**18–20**	(1.00)			(1.00)		
**21–25**	1.361	0.625–2.963	0.438	0.468	0.250–0.873	0.017
**26–30**	1.759	0.733–4.226	0.206	0.597	0.369–0.965	0.035
**31–35**	1.479	0.630–3.475	0.369	1.167	0.716–1.903	0.535
**36–40**	1.304	0.534–3.186	0.561	1.219	0.755–1.967	0.418
**41–45**	1.714	0.645–4.552	0.280	0.860	0.529–1.398	0.543
**Place of residency**						
**Beirut**	(1.00)			(1.00)		
**Mount Lebanon**	0.789	0.510–1.220	0.287	2.232	1.432–3.479	< 0.001^*^
**South**	1.536	0.785–3.008	0.210	2.128	1.336–3.390	0.001
**North**	0.304	0.167–0.551	< 0.001^*^	2.882	1.622–5.121	0.000
**Beqaa**	0.248	0.136–0.452	< 0.001^*^	0.971	0.549–1.716	0.919
**Marital Status**						
**Single**	(1.00)			(1.00)		
**Married**	0.842	0.495–1.432	0.525	0.429	0.190–0.972	0.042
**Divorced**	1.956	0.537–7.127	0.309	0.940	0.422–2.094	0.881
**Income per month**						
**Low**	(1.00)			(1.00)		
**Middle**	1.456	0.812–2.611	0.208	0.436	0.125–1.529	0.195
**High**	4.212	0.702–25.277	0.116	0.858	0.260–2.832	0.801
**Educational level**						
**High school and below**	(1.00)			(1.00)		
**Above high school**	1.201	0.765–1.884	0.427	0.754	0.562–1.012	0.060
**Smoking**						
**No**	(1.00)			(1.00)		
**Yes**	0.362	0.236–0.558	< 0.001^*^	1.440	1.094–1.896	0.009
**Reason for OCP intake**						
**Contraception**	(1.00)			(1.00)		
**Menstrual disorders**	0.263	0.156–0.444	< 0.001^*^	3.051	1.861–5.002	< 0.001^*^
**PCOS**	0.324	0.135–0.773	0.011^*^	0.602	0.358–1.011	0.055
**Duration of OCP intake**						
**Less than or equal to 6 months**	(1.00)			(1.00)		
**More than 6 months**	1.572	1.086–2.275	0.017^*^	0.575	0.444–0.745	< 0.001^*^
**Type of Oral Contraceptive**						
**POP**	2.802	1.770–4.435	< 0.001^*^	9.727	6.821–13.871	< 0.001^*^
**COC**	(1.00)			(1.00)		
**Depression**						
**No**	(1.00)			(1.00)		
**Yes**	14.784	9.035–24.193	< 0.001^*^	15.297	10.144–23.068	< 0.001^*^

Variables entered in the model: age, region, marital status, income per month, educational level, smoking, the reason for OCP intake, duration of OCP intake, type of OCP, and depression degree

1.00 = reference categories

*%* percentage, *OCP* oral contraceptive pill, *COC* combined oral contraceptive, *POP* progesterone only pill, *PCOS* polycystic ovarian syndrome, *CI* confidence interval

^*^Significant at *p*-value < 0.05, *p*-values obtained from logistic regression analysis

In terms of OCPs intake, we also found that using OCPs for conditions other than contraception, such as correcting menstrual irregularities or for PCOS, decreased anxiety, with an adjusted odds ratios of 0.263 (95% CI: 0.156–0.444) *p*-value < 0.001 and 0.324 (95% CI: 0.135–0.773) *p*-value 0.011 respectively. Furthermore, an increase in the duration of OCP intake was associated with an increase in anxiety, with an adjusted odds ratio of 1.572 (95% CI: 1.086–2.275) *p*-value 0.017.

Significantly, the type of OCP was strongly associated with anxiety, with POP representing 2.8 times more risk compared to COCs adjusted odds ratio 2.802 (95% CI: 1.770–4.435) *p*-value < 0.001. Additionally, a notable association was observed between anxiety and depression, with an adjusted odds ratio of 14.784 (95% CI: 9.035–24.193) *p*-value < 0.001. This implies that women on OCPs who were experiencing depression were 14.8 times more likely to develop anxiety.

### The prevalence of depression and its association with the type of oral contraceptive

**Fig. 2:**
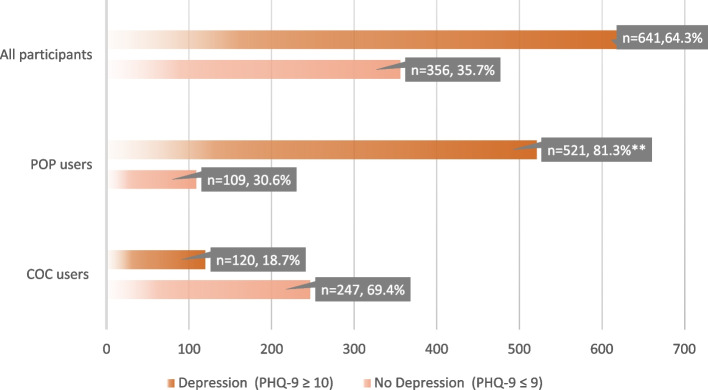
Depression prevalence among All participants, POP users, and COC users. ** Significant at *p*-value < 0.001. COC: combined oral contraceptive, POP: progesterone-only pills. PHQ-9: Patient Health Questionnaire 9

Based on PHQ-9 cut-off scores (≥ 10), the prevalence of depression was 64.3% (Fig. 2), and it was higher among participants taking POP at 81.3% as compared to COC users at 18.7% (Fig. 2).

### The association between depression and different population characteristics

Similarly, the bivariate analysis revealed strong associations between various factors and depression among the participants. Age, current residency, marital status, income per month, educational level, smoking, reason for OCP intake, type of oral contraceptive and anxiety were found to be associated with depression *p*-value < 0.05, indicating their potential as a risk or protective factors. Depression didn’t differ statistically with alcohol consumption, number of children, last delivery, abortion, and duration of OCP intake (*p*-value > 0.05) (Table 4).

**Table 4:** Association between depression and population characteristics

	**Depression**		***P*-value**
	**No Depression n (%)**	**Depression n (%)**	
**Age**			
**18–20**	46 (12.9%)	38 (5.9%)	< 0.001^*^
**21–25**	87 (24.4%)	144 (22.5%)	
**26–30**	78 (21.9%)	103 (16.1%)	
**31–35**	38 (10.7%)	166 (25.9%)	
**36–40**	70 (19.7%)	125 (19.5%)	
**41–45**	37 (10.4%)	65 (10.1%)	
**Place of residency**			
**Beirut**	179 (50.3%)	217 (33.9%)	< 0.001^*^
**Mount Lebanon**	82 (23.0%)	183 (28.5%)	
**South**	37 (10.4%)	52 (8.1%)	
**North**	37 (10.4%)	84 (13.1%)	
**Beqaa**	21 (5.9%)	105 (16.4%)	
**Marital Status**			
**Single**	187 (52.5%)	176 (27.5%)	< 0.001^*^
**Married**	160 (44.9%)	449 (70.0%)	
**Divorced**	9 (2.5%)	16 (2.5%)	
**Income per month**			
**Low**	26 (7.3%)	94 (14.7%)	< 0.001^*^
**Middle**	324 (91.0%)	542 (84.6%)	
**High**	6 (1.7%)	5 (0.8%)	
**Educational level**			
**High school and below**	78 (21.9%)	182 (28.4%)	0.025^*^
**Above high school**	278 (78.1%)	459 (71.6%)	
**Smoking**			
**No**	272 (76.4%)	397 (61.9%)	< 0.001^*^
**Yes**	84 (23.6%)	244 (38.1%)	
**Alcohol Consumption**			
**No**	343 (96.3%)	618 (96.4%)	0.959
**Yes**	13 (3.7%)	23 (3.6%)	
**Number of children**			
**None**	7 (4.0%)	29 (6.2%)	0.567
**1 child**	29 (16.8%)	76 (16.3%)	
**2 or more children**	137 (79.2%)	360 (77.4%)	
**Last Delivery**			
**None**	8 (4.8%)	29 (6.2%)	0.757
**Less than 3 months**	15 (8.9%)	44 (9.5%)	
**More than 3 months**	145 (86.3%)	392 (84.3%)	
**Abortion**			
**Never**	105 (62.1%)	296 (63.7%)	0.922
**Once**	14 (8.3%)	35 (7.5%)	
**Twice or more**	50 (29.6%)	134 (28.8%)	
**Reason for OCP intake**			
**Contraception**	102 (28.7%)	395 (61.6%)	< 0.001^*^
**Menstrual disorders**	214 (60.1%)	201 (31.4%)	
**PCOS**	40 (11.2%)	45 (7.0%)	
**Duration of OCP intake**			
**Less than or equal to 6 months**	220 (61.8%)	376 (58.7%)	0.333
**More than 6 months**	136 (38.2%)	265 (41.3%)	
**Type of oral Contraceptive**			
**POP**	109 (30.6%)	521 (81.3%)	< 0.001^*^
**COC**	247 (69.4%)	120 (18.7%)	
**Anxiety**			
**No**	327 (91.9%)	272 (42.4%)	< 0.001^*^
**Yes**	29 (8.1%)	369 (57.5%)	

*n* frequency, *%* percentage, *OCP* oral contraceptive pill, *COC* combined oral contraceptive, *POP* progesterone only pill, *PCOS* polycystic ovarian syndrome

^*^Significant at *p*-value < 0.05, *p*-values obtained using Pearson’s chi-square test

Table 5 showed the multivariate logistic regression. Where the region of residency had a significant association with depression. Specifically, individuals living in North Lebanon had an adjusted odds ratio of 5.833 (95% CI: 3.233–10.524) *p*-value < 0.001 compared to those living in Beirut. Married women had an adjusted odds ratio of 1.974 (95% CI: 1.178–3.307) *p*-value 0.01, when compared to single women. Interestingly, high and middle economic levels were identified as protective factors against depression, with an adjusted odds ratios of 0.036 (95% CI: 0.006–0.213) *p*-value < 0.001 and 0.434 (95% CI: 0.226–0.834) *p*-value 0.012, respectively. On the other hand, smoking was found to be a risk factor for depression, with an adjusted odds ratio of 2.298 (95% CI: 1.461–3.614) *p*-value < 0.001.

**Table 5:** Odds ratios with their 95% confidence intervals from the logistic regression analysis for depression

Depression						
	**Adjusted Odds Ratio (OR**_**adj**_**)**	**95% CI**	***P*-value**	**crude Odds Ratio (OR)**	**95% CI**	***P*-value**
**Age**						
**18–20**	(1.00)			(1.00)		
**21–25**	1.710	0.805–3.632	0.163	0.470	0.261–0.848	0.012
**26–30**	0.584	0.253–1.350	0.209	0.942	0.581–1.528	0.809
**31–35**	2.220	0.933–5.285	0.071	0.752	0.456–1.239	0.263
**36–40**	1.221	0.509–2.932	0.654	2.487	1.455–4.249	0.001
**41–45**	1.537	0.600–3.941	0.371	1.016	0.617–1.673	0.949
**Place of residency**						
**Beirut**	(1.00)			(1.00)		
**Mount Lebanon**	2.534	1.582–4.061	< 0.001^*^	0.242	0.242–0.403	< 0.001^*^
**South**	0.635	0.319–1.265	0.196	0.446	0.446–0.763	0.003
**North**	5.833	3.233–10.524	< 0.001^*^	0.281	0.281–0.528	< 0.001^*^
**Beqaa**	3.623	1.829–7.180	< 0.001^*^	0.454	0.454–0.834	0.011
**Marital Status**						
**Single**	(1.00)			(1.00)		
**Married**	1.974	1.178–3.307	0.010^*^	0.529	0.228–1.229	0.139
**Divorced**	1.288	0.349–4.762	0.704	1.579	0.684–3.643	0.285
**Income per month**						
**Low**	(1.00)			(1.00)		
**Middle**	0.434	0.226–0.834	0.012^*^	4.338	1.226–15.353	0.023
**High**	0.036	0.006–0.213	< 0.001^*^	2.007	0.608–6.630	0.253
**Educational level**						
**High school and below**	(1.00)			(1.00)		
**Above high school**	1.468	0.905–2.382	0.120	1.413	1.043–1.916	0.026
**Smoking**						
**No**	(1.00)			(1.00)		
**Yes**	2.298	1.461–3.614	< 0.001^*^	0.502	0.375-.0673	< 0.001^*^
**Reason for OCP intake**						
**Contraception**	(1.00)			(1.00)		
**Menstrual disorders**	1.849	1.080–3.165	0.025^*^	3.442	2.134–5.554	< 0.001^*^
**PCOS**	1.294	0.543–3.088	0.561	0.835	0.523–1.332	0.449
**Type of Oral Contraceptive**						
**POP**	9.165	5.790–14.506	< 0.001^*^	9.838	7.286–13.285	< 0.001^*^
**COC**	(1.00)			(1.00)		
**Anxiety**						
**No**	(1.00)			(1.00)		
**Yes**	14.418	8.797–23.631	< 0.001^*^	15.297	10.144–23.068	< 0.001^*^

Variables entered in the model: age, region, marital status, income per month, educational level, smoking, the reason for OCP intake, type of OCP, and anxiety degree

1.00 = reference categories

*%* percentage, *OCP* oral contraceptive pill, *COC* combined oral contraceptive, *POP* progesterone only pill, *PCOS* polycystic ovarian syndrome, *CI* confidence interval

^*^Significant at *p*-value < 0.05, *p*-values obtained from logistic regression analysis

In terms of OCP intake, we also noted a significant association between the development of depression and the reason for OCP intake, as well as the type of OCP being taken. Through logistic regression analysis, it was found that using OCPs to correct menstrual irregularities increased the level of depression, with an adjusted odds ratio of 1.849 (95% CI: 1.080–3.165) *p*-value 0.025.

Significantly, the type of OCP was strongly associated with depression, POP representing 9.2 times more risk compared to COC adjusted odds ratio 9.165 (95% CI: 5.790–14.506) *p*-value < 0.001. Furthermore, a notable association was observed between anxiety and the depression, with an adjusted odds ratio of 14.418 (95% CI: 8.797–23.631) *p*-value < 0.001. This implies that women on OCPs who were experiencing anxiety were 14.8 times more likely to develop depression.

## Discussion

The findings of our study revealed a significant impact of oral contraceptive use on the mental health and well-being of Lebanese women. Our data indicated a high prevalence of anxiety and depression among Lebanese women using OCPs, 39.9% and 64.3% respectively. Many studies investigated the effect of hormonal contraceptives on depression. For instance, a study conducted by Slap GB showed that women using oral contraceptives had depression prevalence ranging from 16 to 56% in nine out of 12 clinical investigations [23]. Similarly, a study conducted in Saudi Arabia reported a depression prevalence of 30.6% among women using hormonal contraception [14]. However, until recently, no reported studies had specifically examined the association between anxiety and oral contraceptive use, with only one study focusing on the consistency of OCP use among women already suffering from anxiety [24].

Furthermore, our study findings also indicated that POPs had a greater impact on mood disorders compared to COCs. The prevalence of both anxiety and depression was higher among POP users compared to COC users. This aligns with a study published in Harvard Health which found that all forms of hormonal contraception were associated with an increased risk of developing depression, with higher risks associated with the progesterone-only forms [25]. Other studies have also shown that progesterone-only contraceptives can contribute to the risk of developing depression to the extent that mood changes have been cited as a primary reason for discontinuing OCP use [9–11, 14]. However, some studies found no independent effect of OCPs on depressive symptoms, even after adjusting for confounders [26].

Regarding the covariates entered in the multivariate analysis, we noted in our study that people living in rural areas of Lebanon such as North and Beqaa were more likely to develop depression but less likely to develop anxiety, this harmonizes with some studies dealing with this issue showing a higher level of depression in rural areas 6.1% versus 5.2% in one study [27],other stating that: “Depression and anxiety prevalence were higher in urban 26.1% compared to rural areas 24.9%” [28].We think that anxiety and depression don’t always follow the same path, in fact, this needs further studies to determine the brain circuitry of both anxiety and depression separately.We also noted similar results with respect to smoking where on one hand smoking protects against anxiety but on the other hand exacerbates depression, we assume that this can be attributed to a widespread belief that smoking reduces stress and can even be used as a kind of “self-medication” by those who are mentally ill, but on the long term nicotine adversely affects the nervous system increasing the risk of developing mental health problems especially depression [29].Last but not least, a positive association noted between married women and development of depression which also accord with other studies as stated by sultana et al. for example [30]. Finally, as the income per month increases this protects against depression which is somehow logical by reducing stressors and providing social resources.

In fact, it is important to note that, endogenous progesterone is beneficial for mood in most women because it transforms into the neuro-steroid allopregnanolone, which facilitates GABA receptors action. Progesterone has a relaxing impact, which explains why it normally has a calming effect in periods with high progesterone levels (such as during pregnancy and the luteal phase). However, hormonal birth control progestins do not convert to allopregnanolone and do not thereby elevate mood. Besides, a deficiency in the endogenous progesterone could lead to varying anxiety, anger, and rage levels [31].

So, the use of synthetic sex steroids in hormonal contraception has been linked to structural and functional alterations in the brain, which can affect cognitive performance, behavior, personality, and emotion [32]. These effects on the brain and behavior can be attributed to several mechanisms. Firstly, hormonal contraceptives act on estrogen and progesterone receptors in various brain regions. Estrogen facilitates glutamate transmission and promotes plasticity, learning, and memory, while progesterone suppresses the glutamate response and enhances GABAergic neurotransmission. Secondly, hormonal contraceptives can reduce endogenous testosterone levels by increasing sex hormone-binding globulin, resulting in estrogen dominance, and feminizing effects on the brain [33]. Lastly, OCPs can lower serotonin and gamma aminobutyric acid (GABA) levels in the brain by suppressing the metabolism of vitamin B6 and vitamin B12 [34].

### Strengths and limitations

The current study possesses several strengths. To our best knowledge, it is the first study to assess the prevalence of anxiety and depression among Lebanese Women using oral contraceptives. Our findings are consistent with previous research, which enhances the validity and reliability of our results. Moreover, we utilized well-validated and reliable scales, specifically the Arabic versions of the Generalized Anxiety Disorder-7 Questionnaire (GAD-7) and the Patient Health Questionnaire-9 (PHQ-9), to evaluate the levels of anxiety and depression among our participants.

However, we acknowledge several potential limitations in our study. Firstly, there may be a selection bias since the participants were not randomly selected, limiting the generalizability of the findings to populations with different demographic characteristics. Secondly, a potential information bias could arise from self-reporting answers to all the questions. Thirdly, despite conducting multivariate analysis to account for confounding factors, there is still a possibility of residual confounding due to unmeasured variables such as weight, physical exercise, and other factors. Furthermore, the cross-sectional nature of the study can demonstrate only association and not a cause-effect relationship. Therefore, it is not possible to ascertain the causality or temporal relationship concerning the pathways of association between oral contraceptive use and appearance of anxiety or depression which is likely to be bidirectional. Fourthly, the use of self-administered questionnaires may limit study participation to women with certain socio-economic characteristics, especially those with higher education. Finally, the study lacked information about the specific doses of estrogen and progesterone in the different OCP formulations used by the participants. To gain a better understanding of this association in a dose-dependent and type-dependent manner, further investigations are necessary. Future prospective studies, as well as experimental studies, are needed to establish a stronger causal relationship and provide more comprehensive insights into the effects of OCPs on mental health outcomes. By addressing these research gaps, we can improve the overall well-being of women using these contraceptives.

## Conclusion

The prevalence of anxiety and depression among Lebanese women using OCPs is high. Our study demonstrated the differential impact of COCs and POPs on our participants mental health outcomes. These results suggest that the low-dose estrogen content in COCs may help mitigate the level of anxiety and depression among women. Research could potentially lead to the development of new formulations in the market and the establishment of clear guidelines and screening protocols for prescribing oral contraceptives. Therefore, healthcare providers, particularly obstetricians should exercise extra caution when prescribing oral contraceptives to women with a history of anxiety or depression, or those with a significant family history of these conditions. Conversely, it is crucial for psychiatrists to inquire about contraceptive use when assessing women seeking therapy for anxiety and depression, as it can significantly impact their mental health.

## Data Availability

The datasets used and/or analyzed during the current study are available from the corresponding author on reasonable request.

## Abbreviations

CI: Confidence interval
COCs: Combined oral contraceptives
FSH: Follicle-stimulating hormone
GABA: Gamma aminobutyric acid
GAD-7: Generalized Anxiety Disorder-7 Questionnaire
KSA: Kingdom of Saudi Arabia
LH: Luteinizing hormone
OCs: Oral contraceptives
OCPs: Oral contraceptive pills
OR_adj_: Adjusted odds ratio
POPs: Progesterone only pills
PHQ-9: The Patient Health Questionnaire-9
PCOS: Polycystic ovary syndrome
